# Evaluating the effectiveness of simvastatin in slowing the progression of disability in secondary progressive multiple sclerosis (MS-STAT2): protocol for a multicentre, randomised controlled, double-blind, phase 3 clinical trial in the UK

**DOI:** 10.1136/bmjopen-2024-086414

**Published:** 2024-09-16

**Authors:** James Blackstone, Thomas Williams, Jennifer M Nicholas, Ekaterina Bordea, Floriana De Angelis, Alessia Bianchi, Alberto Calvi, Anisha Doshi, Nevin John, Sean Apap Mangion, Charles Wade, Rachel Merry, Gil Barton, Dawn Lyle, Elisabeth Jarman, Don Mahad, Abdullah Shehu, Tarunya Arun, Gavin McDonnell, Ruth Geraldes, Matthew Craner, Charles Hillier, Jeban Ganesalingam, Leonora Fisniku, Jeremy Hobart, Cord Spilker, Neil Robertson, Seema Kalra, Stefano Pluchino, Sreedharan Harikrishnan, Miriam Mattoscio, Timothy Harrower, Carolyn Young, Martin Lee, Suresh Chhetri, Fayyaz Ahmed, David Rog, Eli Silber, Paul Gallagher, Martin Duddy, Agne Straukiene, Richard Nicholas, Claire Rice, Stuart J Nixon, Judy Beveridge, Annie Hawton, Susan Tebbs, Marie Braisher, Gavin Giovannoni, Olga Ciccarelli, John Greenwood, Alan J Thompson, Rachael Hunter, Sue Pavitt, Owen Pearson, Nikos Evangelou, Basil Sharrack, Ian Galea, Siddharthan Chandran, Helen L Ford, Chris Frost, Jeremy Chataway

**Affiliations:** 1 Comprehensive Clinical Trials Unit, University College London, London, UK; 2 Queen Square Multiple Sclerosis Centre, Department of Neuroinflammation, UCL Queen Square Institute of Neurology, Faculty of Brain Sciences, University College London, London, UK; 3 Medical Statistics, London School of Hygiene & Tropical Medicine, London, UK; 4 Anne Rowling Regenerative Neurology Clinic, NHS Lothian, Edinburgh, UK; 5 University Hospital Southampton NHS Foundation Trust, Southampton, UK; 6 University Hospitals Coventry and Warwickshire NHS Trust, Coventry, UK; 7 Belfast City Hospital Health and Social Services Trust, Belfast, UK; 8 Oxford University Hospitals NHS Foundation Trust, Oxford, UK; 9 University Hospitals Dorset NHS Foundation Trust, Poole, UK; 10 University Hospitals Sussex NHS Foundation Trust, Brighton, UK; 11 University Hospitals Plymouth NHS Trust, Plymouth, UK; 12 Bradford Teaching Hospitals NHS Foundation Trust, Bradford, UK; 13 University Hospital of Wales, Cardiff, UK; 14 University Hospitals of North Midlands NHS Trust, Stoke-on-Trent, UK; 15 Cambridge University Hospitals NHS Foundation Trust, Cambridge, UK; 16 University of Cambridge, Cambridge, UK; 17 East Kent Hospitals University NHS Foundation Trust, Canterbury, UK; 18 Barking Havering and Redbridge University Hospitals NHS Trust, Romford, UK; 19 Royal Devon University Healthcare NHS Foundation Trust, Exeter, UK; 20 The Walton Centre NHS Foundation Trust, Liverpool, UK; 21 Institute of Systems Molecular and Integrative Biology, University of Liverpool, Liverpool, UK; 22 Norfolk and Norwich University Hospitals NHS Foundation Trust, Norwich, UK; 23 Lancashire Teaching Hospitals NHS Foundation Trust, Preston, UK; 24 Hull University Teaching Hospitals NHS Trust, Hull, UK; 25 Department of Neurology, Salford Royal NHS Foundation Trust, Salford, UK; 26 Department of Neurology, Lewisham and Greenwich NHS Trust, London, UK; 27 Queen Elizabeth University Hospital, Glasgow, UK; 28 Newcastle Upon Tyne Hospitals NHS Foundation Trust, Newcastle Upon Tyne, UK; 29 Torbay and South Devon NHS Foundation Trust, Torquay, UK; 30 Imperial College Healthcare NHS Trust, London, UK; 31 North Bristol NHS Trust, Bristol, UK; 32 MS Society, London, UK; 33 University of Exeter, Exeter, UK; 34 Blizard Institute, Queen Mary University, London, UK; 35 Institute of Ophthalmology, University College London, London, UK; 36 Department of Primary Care and Population Health, University College London Research, London, UK; 37 University of Leeds, Leeds, UK; 38 Swansea Bay UHB, Swansea, UK; 39 Nottingham University Hospitals NHS Trust, Nottingham, UK; 40 Sheffield Teaching Hospitals NHS Foundation Trust, Sheffield, UK; 41 Clinical & Experimental Sciences, University of Southampton Faculty of Medicine, Southampton, UK; 42 Division of Clinical and Surgical Sciences, University of Edinburgh, Edinburgh, UK; 43 Centre for Neurosciences, Leeds General Infirmary, Leeds, UK; 44 National Institute for Health Research, Biomedical Research Centre, University College London Hospitals, London, UK

**Keywords:** Multiple sclerosis, Clinical Trial, Drug Therapy

## Abstract

**Introduction:**

There remains a high unmet need for disease-modifying therapies that can impact disability progression in secondary progressive multiple sclerosis (SPMS). Following positive results of the phase 2 MS-STAT study, the MS-STAT2 phase 3 trial will evaluate the efficacy and cost-effectiveness of repurposed high-dose simvastatin in slowing the progression of disability in SPMS.

**Methods and analysis:**

MS-STAT2 will be a multicentre, randomised, placebo-controlled, double-blind trial of participants aged between 25 and 65 (inclusive) who have SPMS with an Expanded Disability Status Scale (EDSS) score of 4.0–6.5 (inclusive). Steady progression rather than relapse must be the major cause of increasing disability in the preceding 2 years.

Participants will be allocated to simvastatin or placebo in a 1:1 ratio. The active treatment will be 80 mg daily, after 1 month at 40 mg daily. 31 hospitals across the UK will participate.

The primary outcome is (confirmed) disability progression at 6 monthly intervals, measured as change from EDSS baseline score. Recruitment of 1050 participants will be required to achieve a total of 330 progression events, giving 90% power to demonstrate a 30% relative reduction in disability progression versus placebo. The follow-up period is 36 months, extendable by up to 18 months for patients without confirmed progression.

Clinician-reported measures include Timed 25 Foot Walk; 9 Hole Peg Test; Single Digit Modalities Test; Sloan Low Contrast Visual Acuity; Relapse assessment; modified Rankin Scale and Brief International Cognitive Assessment For Multiple Sclerosis. Patient-reported outcomes include MS-specific walking, fatigue and impact scales. A health economic analysis will occur.

**Ethics and dissemination:**

The protocol was approved by the London-Westminster REC (17/LO/1509). This manuscript is based on protocol version 8.0, 26 February 2024. Trial findings will be disseminated through peer-reviewed publications and conference presentations.

**Trial registration numbers:**

NCT03387670; ISRCTN82598726.

**STRENGTHS AND LIMITATIONS OF THIS STUDY**

- The MS-STAT2 trial will be the first phase 3 randomised controlled trial to assess the effectiveness of repurposed simvastatin compared with placebo in slowing the progression of disability in secondary progressive MS as a potential neuroprotective agent.
- It will be the largest ever investigator-initiated phase 3 trial in progressive MS.
- There will be a range of trial sites from neuroscience centres to district general hospitals.
- It will have a relatively high age range (up to 65 years) to increase inclusivity with significant patient and public involvement.
- A limitation is those who are required to be on a statin for their general health are ineligible to participate.

## Introduction

 Multiple sclerosis (MS) is the most common acquired disabling neurological disease affecting adults in temperate latitudes. It is a progressive disorder of the brain and spinal cord (central nervous system) and with current figures (2023) recording a prevalence of 135 000 in the UK and 2.9M globally.^1^

Secondary progressive MS (SPMS) is characterised by a range of severe problems affecting walking, balance, manual function, vision, cognition, pain, bladder and bowel function.^2^,^4^ Rates of conversion from relapsing-remitting MS (RRMS) to SPMS vary depending on definitions used, access to disease-modifying therapy (DMT) and the time period observed. Estimates vary on the risk of conversion, but a recent study reported that the median time from RRMS onset to SPMS is around 30 years^5^ while a separate meta-analysis of SPMS prevalence estimated this would affect up to 50 000 individuals in the UK.^6^ SPMS also has significant financial costs for the National Health Service (NHS), patients and their caregivers; in the UK, the estimated mean annual cost per patient from the societal perspective ranges between £22 000 (Expanded Disability Status Scale, EDSS 4–6.5) and £34 000 (EDSS 7–9).^7 8^

Unlike RRMS, where there is an increasing number (over 15) of DMTs, therapeutic options for those with SPMS are very limited.^9 10^ Siponimod is the only commonly used DMT licensed in the UK for SPMS, but its use is restricted to patients with recent activity (patients with recent inflammatory disease activity, characterised by relapses or new/enhancing MRI lesions). There is a clear lack of effective treatments to impact disease progression.^11^

The MS-STAT phase 2 trial randomised 140 people with SPMS to receive high-dose simvastatin (80 mg/day) or placebo for 2 years. It showed that simvastatin was safe, well tolerated and reduced the rate of annualised brain atrophy by 43% over 2 years. This was supported by positive effects on some secondary outcomes, including clinician-reported (EDSS) and patient-reported (Multiple Sclerosis Impact Scale-29 v2, MSIS29v2) measures.^12^

Despite previous experimental data showing that statins possess immunomodulatory effects in MS,^13^,^16^ the MS-STAT trial did not demonstrate any shift towards a more favourable systemic immunological signal,^12^ suggesting that alternative mechanisms prevail. Statins suppress astroglial and vascular activation,^17^,^19^ improve vascular perfusion and function,^20^,^22^ and attenuate oxidative damage which protects the brain from chronic hypoxic stress. This is especially germane considering evidence that dysfunctional/reduced blood flow in MS may predispose the tissue to damage.^23^,^27^ In support of this, patients with later-stage MS exhibit vascular^28^,^30^ and brain parenchymal cell dysfunction.^1931^,^33^ It is also clear that there is an increase in prevalence of vascular disease in MS, which impacts on disease severity.^34 35^

Therefore, while the cholesterol-dependent actions of statin treatment have been implicated in the past, most evidence continues to support a predominant role for cholesterol-independent processes. Indeed, using a modelling approach from the MS-STAT trial, it was predicted that the beneficial effect was independent of the reduction in total systemic cholesterol levels, signifying the involvement of upstream intermediate metabolites.^36^ Interestingly, a recent Mendelian Randomisation study, looking at the risk of developing MS, implicated the Rho GTPase pathways,^37^ felt to be central to earlier mechanistic animal work.^38 39^

## Methods and analysis

### Trial objective

Following the promising results of the MS-STAT phase 2 trial,^12^ the MS-STAT2 trial has been designed to determine whether simvastatin can slow the time to confirmed disability progression (CDP), based on the current regulatory standard of confirmed EDSS progression.^40^ The primary objective is to compare the effect of daily use simvastatin (80 mg) versus placebo on disability progression at 6 monthly intervals in patients with SPMS, based on change in EDSS score compared with baseline.

### Trial design and setting

**Figure 1:**
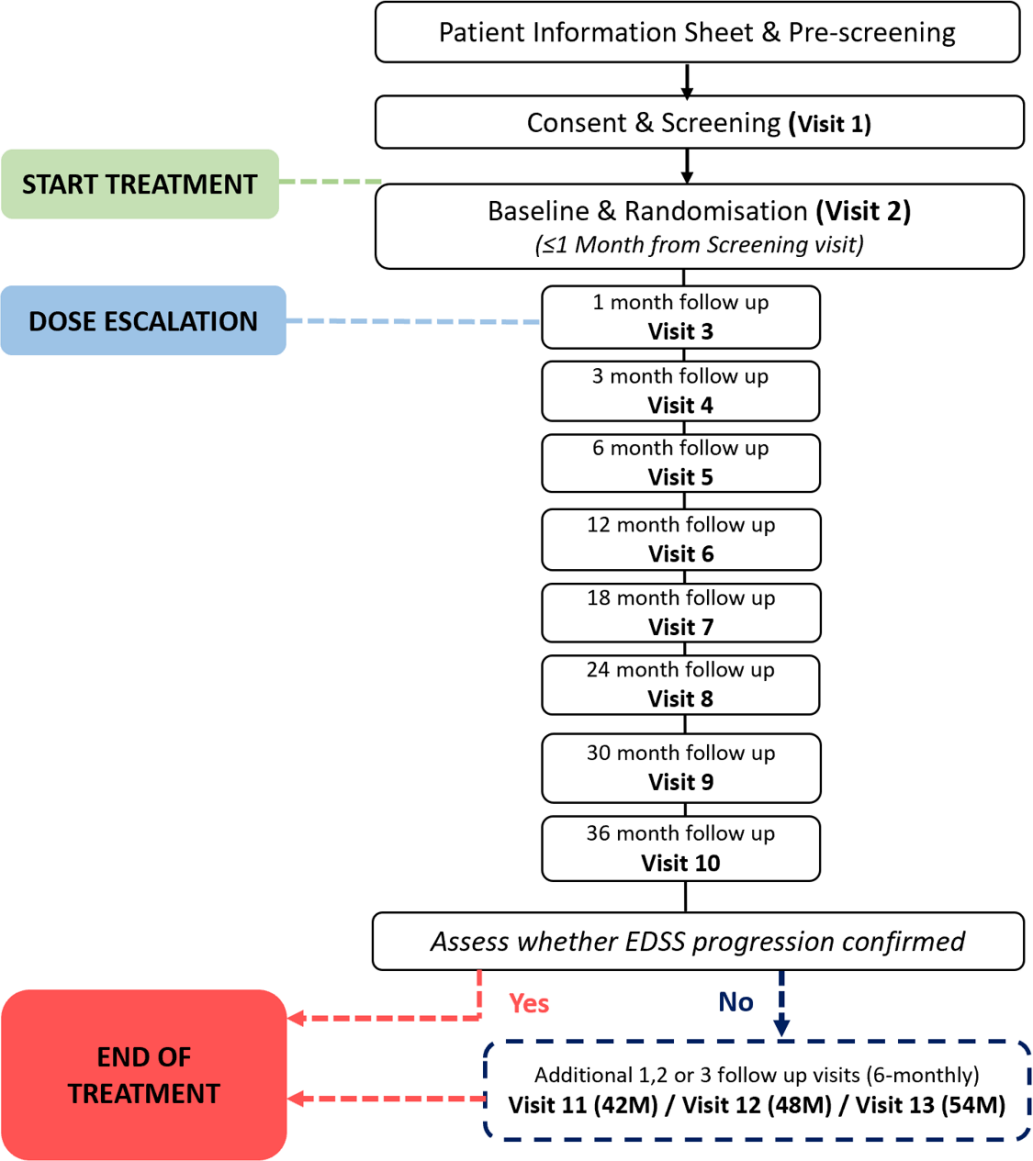
Participant timeline. EDSS, Expanded Disability Status Scale.

MS-STAT2 will be a randomised, multicentre, double-blinded, prospective, parallel-group trial design. The intervention will be oral simvastatin 80 mg vs placebo for patients with SPMS. Figure 1 illustrates the participant pathway. Online supplemental table 1 outlines the trial activities to be conducted, including the clinician and patient-reported outcomes that will be collected. The trial will be conducted at 31 UK NHS hospitals, a mix of trial sites from neuroscience centres to district general hospitals. Participating hospitals will be geographically spread between England, Scotland, Wales and Northern Ireland. The trial will commence in May 2018 and conclude in August 2024.

### Eligibility criteria

Participants will be aged between 25 and 65 years (inclusive) who fulfil the revised McDonald criteria for MS,^41^,^43^ and have SPMS.^44 45^ Steady progression rather than relapse must be the major cause of increasing disability in the preceding 2 years. The inclusion and exclusion criteria are set out in box 1.

Box 1 **Inclusion and exclusion criteria**

**Inclusion criteria**

Patients with a confirmed diagnosis of multiple sclerosis (MS) who have entered the secondary progressive stage. Steady progression rather than relapse must be the major cause of increasing disability in the preceding 2 years. Progression can be evident from either an increase of at least one point if Expanded Disability Status Scale (EDSS) baseline score <6, or an increase of 0.5 point if baseline EDSS score is ≥6, or clinical documentation of increasing disability.

EDSS 4.0–6.5 (inclusive).

Aged 25–65 years old.

Patients must be able and willing to comply with the terms of this protocol.

Provide written informed consent.

**Exclusion criteria**

Relapse within 3 months of baseline visit. Patients will be eligible where 3 months from the final day of the relapse, have elapsed by the date of the baseline visit.

Patients who have been treated with steroids (intravenous and/or oral) due to MS relapse/progression within 3 months from the final day of relapse to the baseline visit. These patients may undergo a further screening visit once the 3-month window has expired and may be included if no steroid treatment has been administered in the intervening period.

(Patients on steroids for another medical condition may be included in the trial provided the steroid prescription is not for MS relapse/progression).

Significant organ comorbidity, for example, cardiac failure, renal failure, malignancy.

Screening levels of alanine aminotransferase/aspartate aminotransferase or creatine kinase ≥3×upper limit of normal.

Current use of a statin; or any use within the last 6 months.

Medications that interact unfavourably with simvastatin as outlined in the current summary of product characteristics (Summary of Medicinal Product Characteristics); including but not limited to CYP3A4 inhibitors (eg, itraconazole, ketoconazole, posaconazole, voriconazole, fluconazole, HIV protease inhibitors (eg, nelfinavir), boceprevir, erythromycin, clrithromycin, telithromycin, telaprevir, nefazodone, fibrates (including fenofibrates), nicotinic acid (or products containing niacin), azole antifungal preparations, macrolide antibiotics, protease inhibitors, verapamil, amiodarone, amlodipine, gemfibrozil, ciclosporin, danazol, diltiazem, rifampicin, fusidic acid, elbasvir, grazoprevir, ticagrelor, daptomycin, grapefruit juice or alcohol abuse.

Primary progressive MS.

Diabetes mellitus type 1.

Uncontrolled hypothyroidism.

Female participants who are pregnant or breast feeding. Women of childbearing potential who are unwilling or unable to use an acceptable method to avoid pregnancy for the entire study period, and up to 4 weeks after the last dose of study drug.

Use of immunosuppressants (eg, azathioprine, methotrexate, ciclosporine) or disease-modifying therapies (avonex, rebif, betaferon, glatiramer) within the previous 6 months.

Use of mitoxantrone, natalizumab, alemtuzumab, daclizumab or other monoclonal antibody treatment, if treated within the last 12 months.

Use of fingolimod, dimethyl fumarate, teriflunomide, cladribine within the last 12 months.

Use of other experimental disease-modifying therapy within the last 6 months.

Commencement of fampridine ≤6 months from the day of randomisation.

Concurrent participation in another clinical trial of an investigational medicinal product or medical device.

Patients with rare hereditary problems of galactose intolerance, the Lapp lactase deficiency or glucose-galactose malabsorption.

### Recruitment and consent

Participating sites will be centres of expertise in MS and trials in MS; have long-term links with the local MS patient population and will have records of patients who may be eligible to participate.

A national-level registration of interest website will be set up to allow patients to register their interest in the trial, and these details will be transferred to the relevant site trial team for further contact.

In prescreening patients (usually by telephone), sites will provide prospective recruits with the trial’s patient information sheet, and then schedule a face-to-face clinic meeting if the patient expresses interest and appears to meet the eligibility criteria.

At a screening visit, participants will be informed of the aims, methods, benefits and potential risks of involvement in the trial and give documented and recorded informed consent prior to any trial-specific procedures being carried out.

Participants are permitted to either be taking newly licensed DMTs at the time of trial commencement (ie, siponimod) or to begin such use as a concomitant medication while in trial.

A lipid profile will be completed at screening only.

### Treatment allocation

Participants will be randomised in a 1:1 ratio between simvastatin or placebo, based on a minimisation algorithm that incorporates the following factors: sex (male/female); age (< or ≥45 years); EDSS baseline score (≤5.5 or ≥6); whether participants are taking newly licensed (2017 onward) DMTs for SPMS; and trial site. An independent and secure online randomisation service (SealedEnvelope.com) will be used by delegated site staff members to directly allocate participants to a treatment arm. Descriptive information (month and year of birth, initials and trial identifier) will be also entered to ensure randomisation allocation matches the intended participant.

### Blinding

All participants, site investigators (including pharmacy) and the trial sponsor team will be blinded to treatment allocation. Only the randomisation service and trial statisticians have access to unblinded treatment allocations during trial recruitment and follow-up.

On randomisation, a five-digit code will be issued to site staff that identifies a blinded bottle of either simvastatin or placebo according to the participant’s allocation. A hard copy of the code will be taken with the prescription to site pharmacy for direct issuance to the participant. Subsequent bottles (‘maintenance kits’) of simvastatin or placebo will be ordered using the same online service and issued to participant on the same basis as at randomisation.

It is not possible to ascertain from the issuance of kit codes which treatment arm a participant, or group of participants, at any site, is on. Patients will be unblinded as to their treatment arm following the final database lock for trial analysis. There will be a procedure in place for emergency unblinding.

## Intervention

### Form

Oral Sandoz simvastatin 40 mg film-coated tablets or matching placebo.

### Dosage

Participants will receive either simvastatin 80 mg (initially 40 mg at randomisation, then escalated after 1 month) or the same quantity of placebo (of the same colour/size), taken once daily at night.

### Duration

Participants will continue on their allocated treatment arm for between 36 and 54 months. Those who have not experienced CDP by the 36-month point will be offered a further period of follow-up, if they are at a site participating in the trial extension period (see the ‘Discussion’ section). Participants in the extension period will remain on their blinded intervention for a further 6–18 months beyond the 36-month time point, dependant on the time remaining before the end of overall trial follow-up. Participants who discontinue treatment will continue to be followed up unless they withdraw consent from participation in the trial.

### Dose modification and stopping

The dose may be varied for patients experiencing hepatic effects, myopathy/rhabdomyolysis or other adverse events at the principal investigator’s discretion. Where elevated alanine aminotransferase (ALT), aspartate aminotransferase (AST) or creatine kinase (CK) levels are sustained at ≥3×upper limit of normal (ULN), it is recommended that the dose is lowered (from 80 mg (active/placebo) to 40 mg (active/placebo)), with rechallenge at the higher dose possible after parameters return to normal. For ALT, AST or CK levels ≥5×ULN, trial medication should be stopped, with rechallenge only permissible where the intervention was clearly determined to be non-causally related (eg, another intercurrent medication or infection, which has now resolved, produced the biochemical abnormalities).

### Outcomes

#### Primary outcome

The primary outcome will evaluate the effect of daily use of simvastatin (80 mg) versus placebo on disability progression at 6-monthly intervals in patients with SPMS, based on change in EDSS score compared with baseline. Progression of disability will be defined as an increase of at least 1 point if the baseline EDSS score <6, or an increase of 0.5 point if EDSS score ≥6. The initial disability progression event is finalised as positive if the disability is sustained and confirmed ≥6 months later. This allows for disability confirmation to take place if a visit is missed. This is termed CDP. The date of progression (if confirmed) will be the initial date of the progression event. EDSS is generally assessed in person, but there will be capacity for this to be collected remotely via the telephone.^46^ Time to first CDP will be compared between the simvastatin and placebo arms over treatment of up to 54 months. EDSS assessment will be performed by a blinded assessor, in line with the current version of the neurostatus scoring guidance (v04/10.2)

#### Secondary outcomes

Clinician-reported secondary outcomes will include Timed 25 Foot Walk (T25FW); 9 Hole Peg Test (9HPT); Single Digit Modalities Test (SDMT); Sloan Low Contrast Visual Acuity (SLCVA); Relapse assessment; modified Rankin Scale (mRS) and Brief International Cognitive Assessment For Multiple Sclerosis comprising SDMT, California Verbal Learning Test-II (CVLT-II) and Brief Visuospatial Memory Test-Revised (BVMT-R).

Patient-reported outcomes will include MSIS-29v2; Multiple Sclerosis Walking Scale-12 v2 (MSWS-12v2); Modified Fatigue Impact Scale - 21 (MFIS-21); Chalder Fatigue Questionnaire (CFQ).

### Health economic outcomes

For the health economic analysis, the EuroQoL EQ-5D 5 level (EQ-5D-5L) and a bespoke resource use measure (RUM), that is, the Client Services Receipt Inventory (CSRI), will be used.

### Trial schedule

Online supplemental table 1 presents the trial schedule of assessments. Following baseline/randomisation, where a full set of clinical assessments and patient-reported questionnaires will be completed, participants will attend the clinic for safety monitoring and dose escalation at 1 month. At 3 months, participants have a telephone check-up (with safety bloods arranged locally), and then will have further assessment visits at 6-monthly intervals from 6 months postrandomisation onward. Where participants are unable to attend 6-monthly follow-ups (such as due to COVID-19-related issues or participant logistics), a telephone-EDSS assessment will be undertaken,^46^ together with remotely collected patient-reported outcomes and locally performed safety bloods.

In terms of clinician-reported outcomes, the EDSS, 9HPT and T25FW will be performed 6 monthly; the SDMT, SLCVA and mRS annually; and CVLT-II and BVMT-R at baseline and the 36-month visits only. For patient-reported outcomes, the EQ-5D-5L and CSRI will be completed 6 monthly; with the CFQ, MFIS-21, MSIS-29v2 and MSWS-12v2 completed annually.

Participants will be evaluated at the 36-month time point visit to determine whether they have experienced EDSS CDP. Those participants yet to have CDP will be asked to consent to continued follow-up into the extension phase, at sites which agree to take part in the extension, as outlined in the ‘Intervention’ section.

At the lead site (University College London Hospitals Trust) only, additional subsidies will be conducted to investigate MRI, biomarker and optical coherence tomography outcomes.

### Power and sample size

In order to have 90% power to demonstrate a 30% relative reduction in disability progression for simvastatin versus placebo, at the conventional 5% significance level, the trial will require a total of 330 progression events. This will be achieved by recruiting 1050 participants with 525 enrolled on each treatment arm. Individual participant trial duration will be a minimum of 36 months (core) and up to a further 18 months (extension) if there is no confirmed progression event by 36 months. This assumes that the placebo progression rate will be 40% by 36 months and 49% by 54 months^47^,^50^ and allows for 20% loss to follow-up by 36 months and 32% by 54 months (among those who are eligible for the extension). This is equivalent to a progression rate of 8% per 6 months among those who have yet to experience progression.

## Analyses

### Statistical analysis

The primary statistical analysis will be based on all participants as randomised, irrespective of subsequent compliance with allocated treatment (intention-to-treat analysis). A secondary per-protocol analysis will be carried out including only participants who were able to comply (see the ‘Compliance’ section) with simvastatin or placebo treatment.

### Primary outcome

The primary analysis will be a comparison of the time to EDSS initial disability progression (if confirmed ≥6 months later) between the simvastatin and placebo arms. HRs and 95% CIs will be calculated using a Cox proportional hazards model and Kaplan-Meier curves produced. The time scale used for survival analysis will be time since randomisation. Participants will be censored if they die, are lost to follow-up, withdraw from the trial or at the final patient visit at which confirmed progression could take place. The model will allow for between-centre variability by stratification by site (baseline hazard allowed to differ by site). In addition, other variables included in the minimisation process will be included as fixed effects. The assumptions underlying the Cox model will be assessed and if there is clear non-proportionality, HRs will be presented separately for the relevant time periods.

### Secondary outcomes

The proportions of participants with confirmed progression at 36 months on the composite (multicomponent) disability progression outcome (EDSS, T25FW or 9HPT), and proportions with confirmed progression on the individual outcomes making this composite, will be compared between arms using logistic regression adjusting for the minimisation variables as fixed effects. Proportions with progression on mRS at 36 months will also be compared using logistic regression, adjusting for the minimisation variables. ORs and 95% CIs will be presented along with the p value for a Wald test for the difference between treatment groups.

Baseline to 36 months change in continuous clinician and patient-reported outcomes will be compared between groups using linear mixed models for repeated measures. For each outcome, the difference in mean of the outcome at 36 months will be reported alongside 95% CIs and the two-sided p value. Analysis of MSIS-29v2, MSWS-12v2, MSFIS-21 and CFQ will use data recorded at all available visits. Analysis of MSFC, T25FW, 9HPT, SLCVA, CVLT-II, BVMT-R and SDMT will use data recorded at baseline and 36 months (due to COVID-19 as described below). The dependent variable will be the outcome at each time point. Treatment group will be included as predictor, with treatment by visit interaction, but at baseline the treatment effects will be constrained to be zero, which is essentially equivalent to adjusting for baseline using analysis of covariance (ANCOVA).^51^ An unstructured covariance matrix for the residuals will be used to allow for correlation between repeated measures on an individual. The minimisation variables, and their interactions with visit, will be included as fixed effects. If parametric assumptions for the linear regression model are substantially violated, bias-corrected and accelerated bootstrap confidence intervals will be used for inference.

The relapse rate up to 36 months will be compared between groups using negative binomial regression adjusting for the minimisation variables as fixed effects. Numbers of relapses by grade, adverse events, serious adverse events and notifiable adverse events will be tabulated by treatment group.

### Compliance

Participants will be considered to be compliant with their randomised intervention if the summary measure of compliance is over 90%, which means they took 80 mg/2 tablets on at least 90% of days over the first 36 months of follow-up, or until the date of confirmed progression, death or withdrawal if these happened before 36 months. A secondary measure of compliance will consider participants compliant if they took either high (80 mg/2 tablets) or low (40 mg/1 tablet) dose on at least 90% of days.

### Interim analyses

The independent data monitoring committee (IDMC) will consider stopping for safety if there is evidence that the trial treatment is worse than placebo alone with a p<0.01 for all-cause deaths. The IDMC will consider stopping for an efficacy-based p<0.001 for a difference between the treatment groups on the primary outcome of 6 months confirmed EDSS progression. Additionally, the IDMC will consider stopping for futility based on recruitment of less than 53% of the final sample size at 15 months after starting the trial.

### Missing data

Missing data will be identified and efforts made to obtain these data. Characteristics of a total number of participants withdrawing and reasons for withdrawal will be tabulated by the treatment group and compared with those with complete data. In the event of substantial differences in withdrawal patterns being found, further sensitivity analyses will be carried out to investigate the robustness of the results.

### Analysis plan

A detailed statistical analysis plan has been written and signed off. All statistical tests will use a two-sided p value of 0.05 unless otherwise specified. Statistical analysis will be performed by using Stata v17.0 (StataCorp).

### Impact of COVID-19 on patient characteristics and trial outcomes

The impact of the COVID-19 pandemic on the trial data will be considered by comparing data collected during three periods, which were chosen based on when public health restrictions were in place in the UK:

- Prior to 16 March 2020—before restrictions.
- From 16 March 2020 to 19 July 2021—during restrictions.
- After 19 July 2021—after restrictions were removed.

Baseline characteristics will be tabulated by treatment group and by mode of visit attendance (in-person vs remote) for visits occurring in the three periods identified above.

A sensitivity analysis will be performed for the primary outcome to assess the impact of COVID-19. This will compare progression at 36 months in trial participants who had EDSS collected at both baseline and month 36 in-person visits during time periods when COVID-19-related public health restrictions were not in force.

A sensitivity analysis will also be performed to compare patient-reported outcomes between trial arms using only data from visits at baseline and 36 months that were collected during periods where public health restrictions were not in force.

In addition, subgroup analyses will examine whether there is evidence that the treatment effect on the primary or secondary outcomes differed between the three time periods, by including a period of treatment interaction in the relevant model.

### Health economics analysis

A treatment that slows progression could represent a highly cost-effective use of NHS resources with the high costs of SPMS and very low cost of simvastatin. A cost–utility study will be carried out to assess the incremental cost per quality-adjusted life-year (QALY) gained from the perspective of the NHS and personal social services (PSS). Cost utility will be estimated for the ‘within trial’ period and also for the lifetime of the patient using a model-based approach.

The within-trial economic evaluation will estimate cost-effectiveness of simvastatin for the first 36 months of the trial period. Health service costs associated with SPMS include inpatient admissions (particularly during relapses or intercurrent illness), outpatient contact (including neurology consultants, MS specialist nurses and allied healthcare professionals), and GP and community care (such as physiotherapy and social worker appointments). Patient resource use will be assessed using the CSRI, a self-complete RUM and using patient records. This RUM will be modified according to the needs of people with SPMS and will be administered at baseline and 6-monthly intervals.

QALYs will be estimated, 6 monthly, using the EQ-5D-5L using the area under the curve approach. Utility scores will be calculated using UK-specific tariffs and adjusting for baseline differences in patients in the trial arms if necessary. In addition, given current uncertainties regarding the appropriateness of the EQ-5D-5L for people with SPMS,^52^ the MSIS-29v2,^53^ a condition-specific measure will be considered for estimating QALYs through methods available in the literature.^54^

The primary within-trial analysis will be intention-to-treat analysis and the secondary analysis will be per protocol analysis in line with the main statistical analysis. Results will be estimated as the incremental cost-effectiveness ratio (ICER) where data will be drawn as far as possible from the trial. Within-trial CIs for mean costs and QALYs will be calculated using a non-parametric bootstrap with replacement.^55^ The results of the non-parametric bootstrap will be presented on a cost-effectiveness plane. The bootstrap replications will be used to construct a cost-effectiveness acceptability curve, which will show the probability that the intervention is cost-effective for different values of the NHS’ willingness to pay for an additional QALY. Appropriate methods for dealing with missing trial data such as multiple imputation will be applied. We will assess the impact of COVID-19 on the ICER in the same fashion as the main statistical analysis.

The decision analytical model will take the form of a Markov model and estimate costs and benefits over the lifetime horizon of the patient to capture the progression of the condition beyond the trial period. The model will include EDSS states and a death state. A secondary analysis from a societal perspective will be undertaken which will consider additional costs borne by the patient such as time off work. Data to populate the model will be obtained from the trial and from published sources. Long-term costs and health outcomes will be discounted using discount rates recommended by the UK National Institute for Health and Care Excellence.^56^

All analyses will conform to recommended economic evaluation methods.^56^ All methods will be described in a detailed health economic analysis plan.

## Monitoring

### Oversight

A trial steering committee and independent data monitoring committee will be formally responsible for the oversight of the trial and ensuring it is conducted in compliance with Good Clinical Practice (GCP), and the relevant regulatory and ethics committee approvals.

The trial management group (TMG) will be responsible for the execution of the trial.

UCL is the sponsor for the trial, with delegated authority to the UCL Comprehensive Clinical Trials Unit (UCL CCTU).

### Safety event reporting

Site teams will report to sponsor all adverse events (irrespective of severity, causality or expectedness in relation to the trial intervention), which will take place from the time of commencement of the intervention to end of patient pathway. Adverse events meeting the ‘serious’ threshold (SAEs) will be subject to expedited reporting to sponsor (immediately and no later than 24 hours from site awareness) in line with the relevant UK regulations.

Additionally, notifiable adverse events (NAEs) for hepatotoxicity or myalgia (supported by laboratory abnormalities of ≥3 times ULN for ALT/AST, respectively) are reportable to the sponsor within the same timelines as for SAEs. Clinical review of all SAEs and NAEs will be undertaken by the sponsor, with events classified as serious adverse reactions (SARs) or suspected unexpected serious adverse reactions (SUSARs) will be onward reported in line with the relevant UK regulations and ethical approvals.

Expectedness will be determined using Section 4.8 of the approved Summary of Medicinal Product Characteristics (SmPC) for simvastatin.

Pregnancy is not an SAE, but following the initiation of the trial medication, if a female participant becomes pregnant, or female partner of a male participant becomes pregnant, then a trial pregnancy notification form will be sent to the sponsor immediately. Pregnancies will be followed up; untoward outcomes will be assessed against trial safety reporting criteria.

### Audit

Clinical trial monitoring will be conducted by the trial coordinating team utilising both a ‘central’ monitoring approach combined with risk-adaptive ‘on-site’ monitoring at NHS clinical and pharmacy sites. Central data review will be used to trigger additional on-site visits where appropriate, but as a minimum, all sites will be monitored in person at least once with thresholds established for planned increase of frequency (eg, proposed site sample size). Central monitoring will be performed by the data manager, and on-site monitoring (including source data verification) will be undertaken by the trial manager, both based at the UCL CCTU. A monitoring plan will be created and updated at least annually, with UCL CCTU Quality Management Group oversight.

### Emergency contacts and unblinding

All recruited participants will be given a card with contact details of the clinical trial team including an emergency contact available out of hours. In the event unblinding becomes necessary, this will be performed through the 24 hours, 7 days a week web-based service offered by the trial’s randomisation service. It is expected that emergency unblinding will only be performed for those patients experiencing a medical emergency for which the clinical management will require knowledge of the participant’s treatment allocation. It is anticipated that for the majority of clinical care instances, appropriate management can proceed with the assumption that the patient has been treated with simvastatin without needing to unblind the participant.

### Withdrawal of trial participants

Participants may withdraw from trial participation at any time without prejudicing their right to NHS standard of care. Participants who discontinue the trial intervention will remain in follow-up unless they withdraw consent. Data from withdrawn participants will be included in the analysis unless they specifically withdraw consent for its use.

### Patient and public involvement

MS-STAT2 has a strong patient and public involvement (PPI) strategy with significant contributions from the UK Multiple Sclerosis Society (UK MSS) PPI representatives and members of the UK MSS-PPI Forum to maximise patient benefit. Essential feedback provided on factors that could have an impact on participation such as age, entry disability, trial schedule and disability fluctuation have been taken into consideration and have been embedded in the protocol to ensure that it is acceptable to the patient community. PPI input will facilitate retention in the trial.

The UK MSS and forum will work closely with the research team to maximise participant retention by codeveloping a tailored communication strategy including making use of the existing UK MSS programme of events and publications to promote the trial to people living with MS. They will also explain the importance of minimising drop-out and encouraging UK MS Register enrolment.

Highly experienced PPI representation is also embedded into the operational functioning of the trial, through membership of both the trial management group and trial steering committees.

### Ethics and dissemination

#### Data management and confidentiality

All trial documentation at site will be held in restricted access areas and stored securely by the site trial teams. Trial data will be initially entered onto paper case report forms (CRFs) and then entered by sites into a secure, validated online database (Elsevier MACRO V.4.0) and accessible only by delegated team members of that site, and by delegated staff from the coordinating centre at UCL CCTU.

CRFs for the trial will identify participants using a unique five-digit trial identifier, month and year of birth and initials. Under the UK Data Protection Act 2018, the latter identifiers will be considered as personally identifying and will be treated as such by both the site team and coordinating centre team.

Where written communication (eg, data queries) on individual patient cases is necessitated between sites and the coordinating centre, only the trial identifier should be used in the first instance.

Any transfer of documentation containing personally identifying data between site and coordinating centre will be subject to AES-256 industry-standard encryption.

###  Patient consent

All MS-STAT2 participants will need to provide informed consent as per the principles of GCP (as defined in the UK Medicines for Human Use (Clinical Trials) Regulations SI 2004) prior to any trial-specific procedures being undertaken. Participants will also agree to sponsor review of their medical records for clinical trial monitoring or inspection purposes.

###  Ethical approval and dissemination

The National Research Ethics Service Committee (London, Westminster) reviewed the trial protocol and materials to be given to patients (approved 9 October 2017, REC ref 17/LO/1509). This article refers to the current protocol (V.8.0, 26 February 2024). Online supplemental appendix 1 lists subsequent protocol amendments with reasons.

The findings of this trial will be disseminated through peer-reviewed publications and conference presentations. There will be a close communication strategy developed with the UK MSS and full PPI engagement.

### Access to data

Data will be stored on an encrypted and password-protected database. Site staff will have direct control of their own site’s data.

Where future analyses are proposed, these will be considered by the trial’s publications committee with appropriate data sharing formal agreements to be put into place.

## Discussion

Several trial design changes have been required since the commencement of trial recruitment in 2018 (see online supplemental appendix 1). We discuss here, two key modifications to facilitate understanding of the Protocol V.8.0 as described above: (1) the addition of an extension period for participants not yet achieving CDP by month 36 and (2) the need to update some trial analyses in light of the COVID-19 pandemic period.

The original trial design allowed for a fixed 36-month period on IMP (with an additional 3-month period allowable for participants with initial disease progression at month 36 to allow for confirmation of such progression). Following permission of the trial steering and independent data monitoring committees, the team amended the protocol in February 2021 to permit a longer duration of follow-up (remaining on blinded trial drug/placebo) for participants who had not had CDP by month 36. The expectation was to capture the necessary number of primary outcome events critical to achieving adequate power on the trial while using a smaller than anticipated sample of patients: 1180 in the original design, revised to 1050 in the revised design, with an extension period of up to 54 months, dependent on the time remaining until last participant visit. The overwhelming majority of sites (n=21) were able to participate in this, though a handful of lower recruiting sites reported lacking additional resource, and several sites commenced recruitment too late to enter participants into the extension period of the patient pathway.

The COVID-19 pandemic in the UK proved challenging for trial recruitment: no recruitment activity was either permitted or viable for several months in the summer of 2020. Moreover, participant access to trial sites was highly restricted, and as a consequence, the ability to be assessed for face-to-face secondary clinical outcomes, particularly T25FW, 9HPT, SLCVA, SDMT, CVLT-II, BVMT-R. Following trial management team review in 2022 of the rates of postbaseline missing data for these outcomes, the decision was taken to move to a binary classification of confirmed progression by month 36 for these variables.

## supplementary material

10.1136/bmjopen-2024-086414 online supplemental file 1

10.1136/bmjopen-2024-086414 online supplemental table 1
