# Supplementary material for: Evaluating the effectiveness of simvastatin in slowing the progression of disability in secondary progressive multiple sclerosis (MS-STAT2): protocol for a multicentre, randomised controlled, double-blind, phase 3 clinical trial in the UK
Source: BMJ Open. 2024 Sep 16;14(9):e086414. doi: 10.1136/bmjopen-2024-086414 (PMC11409264; doi:10.1136/bmjopen-2024-086414)
Supplement: online supplemental file 1 [file bmjopen-14-9-s001.pdf]

APPENDIX 1: Protocol amendments table

Protocol Version	Protocol Date	Summary of Changes
1.0	1- Aug- 2017	N/A
2.0	24-Jan-2018	1. Addition of a new exclusion criteria – Patients with rare hereditary problems of galactose intolerance, the lapp lactase deficiency or glucose-galactose malabsorption may experience a serious reaction to use of simvastastin as each 40mg film-coated tablet contains 116.4 mg lactose per film-coated tablet. Exclusion criteria to be amended to ensure patients with lactose intolerance as a result of rare hereditary problems of galactose intolerance, the lapp lactase deficiency or glucose-galactose malabsorption are not enrolled to the trial<br>2. Inclusion of trial identifiers<br>- ClinicalTrials.gov unique identifier<br>- Clinical Trial Authorisation (CTA) number<br>3. Use of two new questionnaires at all participating sites<br>a. Modified Fatigue Index Scale – 21 (MFIS-21)<br>b. Chalder Fatigue Questionnaire (CFQ)<br>4. Addition of three sub-studies at participating site(s) only<br>- MRI sub-study (Appendix 1)<br>- Biomarker sub-study (Appendix 2)<br>- OCT sub-study (Appendix 3)<br>5. Recruitment of healthy blood donors for the biomarker sub-study<br>6. Change from ABILHAND-56 to ABILHAND-23<br>7. Section 7.5.1 has been revised to outline process of obtaining consent from sub-study participants<br>8. SAE form to be sent to trial team via secure portal/encrypted<br>9. Change in wording – Oversight group changed from Independent Data and Monitoring Committee (IDMC) to Data Monitoring and Ethics Committee (IDMC) in line with funder (NIHR) terminology<br>10. Editing of section 6.11.3.4: Procedures following notification of pregnancy<br>11. Addition of section 6.11.3.4.2 Notification by male participants in the event of partner becoming pregnant<br>12. Addition of new terms to Glossary (section 4)<br>13. Minor edits and formatting throughout the protocol
3.0	02-Aug-2018	1. New logo for UCL CCTU (front page)<br>2. Addition of reference to EU General Data Protection (GDPR) 2016 in section 1.1, 6.10<br>3. Inclusion criteria edited to remove:<br>- that patients must have entered the secondary progressive stage 'at randomisation'<br>- inclusion criteria for being male or female<br>4. Date of first enrolment amended to May 2018 (structured summary section 1.3)<br>5. Addition of CSRI form as a secondary outcome measure (structured summary section 1.3)<br>6. List of sub-studies added to structured summary (section 1.3)<br>7. Protocol contributors (section 1.4.1), Trial sponsor and funders (section 1.4.2), Trial team (section 1.4.3), Trial Management Group (section 1.4.4), Trial Steering Committee (section 1.4.5), Independent Data Monitoring Committee (section 1.4.6), and Recruitment Management Group (section 1.4.6) edited due to staff changes and to add clarity and consistency.<br>8. References to DMEC changed to IDMC throughout<br>9. Inclusion criteria (section 1.3, 6.3.1.2 and 6.5.1) updated to remove reference to screening EDSS<br>10. Addition of new terms to Abbreviations (section 3)

Protocol Version	Protocol Date	Summary of Changes
		11. Addition of new terms to Glossary (section 4)<br>12. Secondary objectives (section 5.2.2.2) updated for clarity, to add CSRI form, be clear which outcomes relate to Health Economics, and to remove SF-36 and ABILHAND-23<br>13. Visit numbers edited to refer to month numbers only (week numbers removed)<br>14. Eligibility criteria for individuals performing the interventions clarified and moved to section 6.5.5.3<br>15. Co-enrolment guidance (section 6.3.1.5) clarified<br>16. Blood samples taken during screening procedures (section 6.3.1.6) clarified<br>17. Clarification added that if screening blood tests are clinically significant these should be repeated prior to the baseline visit (section 6.3.1.6)<br>18. Clarification around re-screening patients under new patient identification numbers added (section 6.3.1.6)<br>19. Drug referred to as 'trial medication' consistently<br>20. Clarification around additional visit 11 added (section 6.4.2 and figure 1)<br>21. Accountability (section 6.4.5) updated to state patients should return all unused trial medication at each visit, and that destruction can occur as per standard local policy<br>22. Compliance and adherence (section 6.4.6) updated to clarify that the diary card will record doses trial medication taken since the last visit<br>23. Concomitant care (section 6.4.7) updated for clarity in relation to statins and DMDs<br>24. Contraindicated medications (section 6.4.8) updated regarding re-starting trial medication after taking a contraindicated medication for a short period of time<br>25. Overdose (section 6.4.9) updated to state that patients who overdose should discontinue trial medication but remain in follow up and not be withdrawn<br>26. Protocol treatment discontinuation (section 6.4.10) updated to state that patients should remain in follow up unless they specifically withdraw consent to do so<br>27. Primary outcome (section 6.5.1) updated to clarify when the EDSS should be done by a treating or assessing clinician<br>28. SLCVA, CVLT-II, BVMT-R (section 6.5.2.1) guidance updated for clarity and to specify which subsets of the CVLT-II and BVMT-R are being completed as part of BICAMS<br>29. Relapse assessment guidance (section 6.5.1) updated to be clear that grade 3 relapses should be reported as an SAE<br>30. Participant timeline (section 6.6) updated to increase the number of timepoints the 9HPT and T25FW completed at<br>31. Participant timeline (section 6.6) updated to reduce the number of timepoints the SLVCA, mRS, MSIS-29v2, MSWS-12v2, EQ-5D-5L and Lipid profile completed at, and to remove the SF-36<br>32. Loss to follow-up (section 6.6.3) updated to include methods to attempt to contact patients before considered lost to follow up, and the use of telephone assessment EDSS and posted patient reported outcome measure for patients that are unable to attend clinic<br>33. Participant timeline (section 6.6) updated to reduce the number of timepoints bloods are taken for the biomarkers sub-study<br>34. Additional footnotes added to participant timeline (section 6.6) for clarity<br>35. Participant timeline (section 6.6) updated to move ABILHAND-23 to sub-study section as now being using only at the lead site (UCLH) as a sub-study<br>36. Participant timeline (section 6.6) additional footnote added regarding timing of baseline MRI scan for MRI sub-study<br>37. Early stopping of follow up (section 6.6.1) updated in regards to intention-to-treat<br>38. Participant transfers (section 6.6.2) updated in regards to responsibility for resolution of data queries<br>39. Loss to follow-up (section 6.6.3) updated to clarify procedures for preventing loss to follow up<br>40. Recruitment (section 6.81) updated to specify when PIS should be provided to potentially interested patients

Protocol Version	Protocol Date	Summary of Changes
		41. Figure 2 updated with a more accurate map of expected sites<br>42. Assignment of intervention (section 6.9) updated to clarify that the patient identification number will be the screening number, and the patient will be randomised under this number. References to drug identification codes replaced with 'kit codes'<br>43. New section 6.9.4 added for unblinding following trial closure<br>44. Data collection methods (section 6.10.1) updated regarding training required for EDSS as the primary outcome measure.<br>45. Data collection methods (section 6.10.1) updated clarifying which documentation is considered as source documentation<br>46. Non-adherence and non-retention (section 6.10.3) updated in regards to how compliance is assessed<br>47. Statistical analysis plan (section 6.10.4.1) updated to clarify that only the IDMC will see the result of interim unblinded analyses<br>48. Analysis population and missing data (section 6.10.5) updated to clarify that compliance will be assessed on reported missed doses<br>49. Health economics (section 6.10.6) sections numbered for clarity<br>50. Health Economics (section 6.10.6.2) updated to remove that an application will be made for Hospital Episode Statistics<br>51. Timing of interim analyses (section 6.11) updated due to annually<br>52. Data monitoring for harm (section 6.11.3) updated in regard to requirements for reporting AEs solely related to MS<br>53. Other notifiable AEs (section 6.11.3.3) updated to include reporting pregnancies for female partners of male participants<br>54. Notification of SAEs by investigators to CCTU (section 6.11.3.6.1) updated to state month and year of birth will be collected not full date of birth<br>55. CCTU responsibilities for clinical review of SAEs (section 6.11.3.6.2) clarified<br>56. Confidentiality (section 7.6) updated to provide additional information<br>57. Appendix 1 MRI sub-study, Appendix 2 Biomarkers sub-study, Appendix 3 OCT sub-study have had eligibility criteria added<br>58. Appendix 1 MRI sub-study eligibility (section 1) state that patients that cannot tolerate gadolinium can still participate without gadolinium<br>59. Appendix 1 MRI sub-study the secondary outcomes (section 5.1.2) have been updated to correct the minimisation variables<br>60. Appendix 1 MRI sub-study secondary outcomes (section 7.1.2) clarified<br>61. Appendix 2 Biomarkers sub-study data collection for healthy donor (section 1.3) updated to state initials not name will be collected<br>62. Appendix 2 Biomarkers sub-study Aims (section 2) updated to specify exactly what samples will be tested<br>63. Appendix 2 Biomarkers sub-study objectives (section 5) clarified<br>64. Appendix 2 Biomarkers sub-study outcomes (section 6) updated to clarify outcomes and include additional exploratory outcome<br>65. Appendix 4 ABILHAND-23 sub-study added
4.0	22-Jul-2019	1. Exclusion criteria updated to exclude patients taking elbasvir, grazoprevir and recent cladribine (structured summary section 1.3, section 6.3.1.3)<br>2. Exclusion criteria updated to state dimethyl fumarate instead of just fumarate (structured summary section 1.3, section 6.3.1.3)<br>3. Structured summary (section 1.3) updated primary outcome should be change in EDSS in comparison to baseline visit (not screening visit)<br>4. Creatinine kinase corrected to creatine kinase throughout<br>5. Laboratory abnormalities (section 6.4.4.1) updated to include further information about myopathy

Protocol Version	Protocol Date	Summary of Changes
		6. Laboratory abnormalities (section 6.4.4.1) updated to provide greater clarity on the dose modification strategy due to AEs and other reasons.<br>7. Contraindicated medications (section 6.4.8) updated to include elbasvir and grazoprevir<br>8. Trial closure (section 6.6.4) definition of end of trial updated<br>9. SLCVA guidance (section 6.5.2.1) updated to state that the SLVCA should only be tested binocularly.<br>10. Participant timeline (section 6.6) updated to remove the FAB. The FAB will only be completed at baseline and visit 10 at UCLH as a separate sub-study<br>11. Participant timeline (section 6.6) footnote B edited to state that safety bloods do not required fasting and to remove potassium and sodium for safety bloods test except at screening<br>12. Map (section 6.8.1) updated to show currently open sites<br>13. Clarification that only AEs which are both non-serious AND can be attributed solely to the progression of the patient's SPMS condition can be excluded from reporting (section 6.11.3.2).<br>14. Section 6.11.3.5.2 updated from CTCAE V4 to CTCAE V5<br>15. Section 6.11.3.5.4 updated in relation to sponsor assessing expectedness for SAEs<br>16. Section 6.11.3.6.1 updated to remove investigator assessing expectedness for SAEs<br>17. Section 6.11.3.5.2 updated in relation to sponsor assessing expectedness for SAEs<br>18. References corrected for minor duplication and entered into Endnote system.<br>19. Appendix 2 Biomarkers sub-study aims (section 2) updated, frozen serum and plasma samples will be stored to allow future analysis of novel biomarkers. Order of paragraphs changed to improve fluidity of reading.<br>20. Appendix 3 OCT sub-study updated to include Sloan Low Contrast (100/2.5/1.25%) assessment for OS/OD (removed from main study for other sites).<br>21. Appendix 5 added for FAB sub-study
5.0	23-Mar-2020	1. Clarification in Section 6.4.3.1 that safety blood testing will be performed at the following visits:<br><br>- Visit 3 (Month 1) to evaluate patient tolerance of low (40mg) IMP dose<br>- Visit 4 (Month 3) to evaluate patient tolerance of high (80mg) IMP dose<br>- Visit 5 (Month 6) to Visit 10 (Month 36) at least annually<br><br>Where a patient cannot attend clinic/GP to receive safety blood evaluation of tolerance of high dose at Visit 4 (month 3), they should be de-escalated to low (40mg) dose, which is already known to be tolerated, and re-challenged subsequently where no clinical reason not to do so.<br><br>Once a patient is established on high (80mg) dose, it is appropriate for patients to have safety blood testing at least annually from this point. Where clinical need is indicated, safety blood testing may be more frequent (e.g. six-monthly).<br><br>Patients will not have additional IMP dispensed to them where it has been ≥12 months since last evaluation of safety bloods.
6.0	11-Aug-2020	1. Exclusion criteria (section 1.3 and section 6.3.1.3) updated to clarify minimum acceptable period since MS relapse<br>2. Exclusion criteria (section 1.3 and section 6.3.1.3) updated to exclude patients taking Ticagrelor and Daptomycin<br>3. Laboratory abnormalities (section 6.4.4.1) updated to include information about concomitant medications Ticagrelor and Daptomycin.

Protocol Version	Protocol Date	Summary of Changes
		4. Dose modification guidance for re-challenging IMP updated (section 6.4.4.2 and 6.4.4.3)<br>5. Concomitant medications Ticagrelor and Daptomycin added to contraindicated medications (section 6.4.8).<br>6. Participant timeline (section 6.6) updated to allow, under exceptional circumstances (i.e. due to COVID-19) the following:<br>- Screening (visit 1) and baseline (visit 2) can be combined<br>- Month 1 (visit 3) can be remote<br>7. Loss to follow-up (section 6.6.3) updated to include categories of trial participation, and related guidance<br>8. Figure 2: Map of expected MS-STAT2 sites (section 6.8.1) updated
7.0	17-Feb-2021	1. Structured summary (section 1.3) updated to reflect changes in the main body of the protocol.<br>2. Details of CCTU trial team, MS Society staff and PPI representation updated (section 1.4).<br>3. Trial diagram updated to include additional visits for certain patients (section 2).<br>4. Updated of abbreviations to include LPLV ('last patient last visit') (section 3).<br>5. Glossary updated to define meaning of last patient last visit (section 4).<br>6. Aim of trial redefined to determine whether rate of disability progression can be slowed over the duration of the treatment period, rather than 3 years as previously (section 5.2.1)<br>7. Primary objective updated to note for those patients requiring a final visit following Visit 10 specifically to confirm their progression event but who lack 6 months until LPLV, can have their visit 3-6 months after Visit 10 should time until LPLV allow (section 5.2.2.1).<br>8. Trial design section updated to allow a series of additional 6-monthly visits for those patients who have not had a confirmed progression event by their Visit 10 (36M). There will be between 1-3 additional visits, dependant on time remaining until LPLV date (section 5.3).<br>9. Treatment schedule updated to include provision that patients are requested to attend additional visits after Visit 10 will remain on IMP between these visits (section 6.4.2).<br>10. Schematic showing drug regimen updated (section 6.3).<br>11. Primary outcome updated to address previous error by noting EDSS scores at follow-up visits will be evaluated against baseline EDSS (not screening EDSS). Wording also updated to confirm where patients have less than 6 months until LPLV but need a confirmatory final visit for an initial progression can do so at 3-6 months from Visit 10, where time until LPLV allows (section 6.5.1).<br>12. Secondary outcome analysis updated to focus on proportion of progression events rather than time to progression (section 6.5.2).<br>13. Participant timeline updated to allow for additional visits beyond 36 months for those patients yet to have a confirmed progression event (section 6.6).<br>14. New section added to explain process of determining whether patients reaching Visit 10 (36M) will be asked to attend additional visits or end their trial pathway at the Visit 10 timepoint (section 6.6.2).<br>15. Sample size updated to explain the decision to reduce the sample size from 1,180 to 1,050 patients – including the potential for reducing further to 950 patients owing to COVID-19 impacts on recruitment. The implications for power on primary outcome are also addressed (section 6.7)<br>16. Statistical methods (outcomes) revised on when the primary outcome censor point will occur (final patient visit where a confirmed progression could occur, rather than 36 months post-baseline). Also proportion rather than time to disability will be used

Protocol Version	Protocol Date	Summary of Changes
		in the secondary outcome analyses, using a mixed effect logistic regression approach, rather than time to event analysis (section 6.10.4.2)<br>17. Additional analyses – adjusted has been updated to add the wording 'or inclusion as a random effect' in relation to centre variability (section 6.10.4.3)<br>18. Analysis population updated to clarify that patient adherence will be assessed until their final visit rather than 3 years (section 6.10.5).
V8.0	26-Feb-2024	1. Structured summary updated to reflect changes throughout the main body of the protocol (section 1.3)<br>2. Staffing names, roles and affiliations updated (section 1.4)<br>3. Background/rationale updated to reflect current UK/global prevalence data, alongside updated patient costs estimates. Additionally, the limited introduction of Siponimod in the UK context is noted. Finally, recent research exploring the potential mechanisms of action for statins are provided.<br>4. The primary objective is updated to remove the option that participants with initial disease progression at v10 can have a confirmatory visit 3-6 months later. Instead, all visits are treated equally in this respect (section 5.2.2.1).<br>5. The secondary objectives have been given additional granularity on analysis approach (section 5.2.2.1).<br>6. In line with the update to the primary objective, wording supporting a confirmatory visit 3-6 months after an initial progression at visit 10 is removed (sections 5.3, 6.4.2, 6.4.3, and 6.5.1, and 6.6 Footnote).<br>7. The expectation that in-person visits should take place at Baseline and Visit 10 is clarified (section 6.6 footnote).<br>8. Further detail is provided around planned statistical subgroup analyses in light of the COVID-19 pandemic in 2020-21 (section 6.10.4.1).<br>9. Correction of a typo that omitted in error the word 'confirmed' in relation to the composite outcome definition in Statistical Methods (section 6.10.4.2).
