# Supplementary material for: Evaluating the effectiveness of simvastatin in slowing the progression of disability in secondary progressive multiple sclerosis (MS-STAT2): protocol for a multicentre, randomised controlled, double-blind, phase 3 clinical trial in the UK
Source: BMJ Open. 2024 Sep 16;14(9):e086414. doi: 10.1136/bmjopen-2024-086414 (PMC11409264; doi:10.1136/bmjopen-2024-086414)
Supplement: online supplemental table 1 [file bmjopen-14-9-s002.pdf]

Supplementary Table. Schedule of assessments

**Clinic visit number**	**VISIT 1**	**VISIT 2**	**VISIT 3**	**VISIT 4**	**VISIT 5**	**VISIT 6**	**VISIT 7**	**VISIT 8**	**VISIT 9**	**VISIT 10**	**VISIT 11***	**VISIT 12***	**VISIT 13***
**Month**	**SCREENING**	**Month 0 BASELINE**	**Month 1**	**Month 3 TELEPHONE**	**Month 6**	**Month 12**	**Month 18**	**Month 24**	**Month 30**	**Month 36**	**Month 42**	**Month 48**	**Month 54**
**Protocol window**		**(≤1m from screening)**	**(+/-1 week)**	**(+/-1 week)**	**(+/- 2 weeks)**	**(+/- 2 weeks)**	**(+/-2 weeks)**	**(+/-2 weeks)**	**(+/-2 weeks)**	**(+/-2 weeks)**	**(+/-2 weeks)**	**(+/-2 weeks)**	**(+/-2 weeks)**
Informed consent	X												
Inclusion/exclusion criteria review	X	X											
Demography, Review of medical & MS history	X												
EDSS – Treating clinician	X												
Physical examination	X		X		X	X	X	X	X	X	X	X	X
Vital signs	X		X		X	X	X	X	X				
Urine pregnancy test	X	X											
Safety bloods	X	X	X	X	X	X	X	X	X	X	X	X	X
Lipid profile, Thyroid function	X												
Compliance assessment			X	X	X	X	X	X	X	X	X	X	X
Relapse assessment (count & grade)	X	X	X	X	X	X	X	X	X	X	X	X	X
Adverse events		X	X	X	X	X	X	X	X	X	X	X	X
Concomitant medication	X	X	X	X	X	X	X	X	X	X	X	X	X
Randomisation		X											
Dispense trial medication		X	X		X	X	X	X	X	X	X	X	
Trial medication - dose escalation			X										
**Clinician reported outcome measures**													
EDSS – Independent Assessing clinician		X			X	X	X	X	X	X	X	X	X
9HPT, T25FW		X			X	X	X	X	X	X	X	X	X
SDMT, SLCVA, mRS		X				X		X		X			
CVLT-II, BVMT-R		X								X			
**Patient reported outcome measures**													
MSIS-29v2, MSWS-12v2, MFIS-21, CFQ		X				X		X		X			
CSRI, EQ-5D 5L		X			X	X	X	X	X	X			

* Visits 11-13 form an ‘extension’ period follow-up for participants who do not have CDP on the EDSS Step score on/by Visit 10.
