# Supplementary material for: Evaluating the effectiveness of simvastatin in slowing the progression of disability in secondary progressive multiple sclerosis (MS-STAT2): protocol for a multicentre, randomised controlled, double-blind, phase 3 clinical trial in the UK
Source: BMJ Open. 2024 Sep 16;14(9):e086414. doi: 10.1136/bmjopen-2024-086414 (PMC11409264; doi:10.1136/bmjopen-2024-086414)
Supplement: Supplementary file 3 [file bmjopen-2024-086414.reviewer_comments.pdf]

PEER REVIEW HISTORY

BMJ Open publishes all reviews undertaken for accepted manuscripts. Reviewers are asked to complete a checklist review form and are provided with free text boxes to elaborate on their assessment. These free text comments are reproduced below.

ARTICLE DETAILS

**TITLE (PROVISIONAL)**	Evaluating the effectiveness of simvastatin in slowing the progression of disability in secondary progressive multiple sclerosis (MS-STAT2): protocol for a multicentre, randomised controlled, double-blind, phase 3 clinical trial in the United Kingdom
**AUTHORS**	Blackstone, James; Williams, Thomas; Nicholas, Jennifer; Bordea, Ekaterina; De Angelis, Floriana; Bianchi, Alessia; Calvi, Alberto; Doshi, Anisha; John, Nevin; Apap Mangion, Sean; Wade, Charles; Merry, Rachel; Barton, Gil; Lyle, Dawn; Jarman, Elisabeth; Mahad, Don; Shehu, Abdullah; Arun, Tarunya; McDonnell, Gavin; Geraldes, Ruth; Craner, Matthew; Hillier, Charles; Ganesalingam, Jeban; Fisniku, Leonora; Hobart, Jeremy; Spilker, Cord; Robertson, Neil; Kalra, Seema; Pluchino, Stefano; Harikrishnan, Sreedharan; Mattoscio, Miriam; Harrower, Timothy; Young, Carolyn; Lee, Martin; Chhetri, Suresh; Ahmed, Fayyaz; Rog, David; Silber, Eli; Gallagher, Paul; Duddy, Martin; Straukiene, Agne; Nicholas, Richard; Rice, Claire; Nixon, Stuart; Beveridge, Judy; Hawton, Annie; Tebbs, Susan; Braisher, Marie; Giovannoni, Gavin; Ciccarelli, Olga; Greenwood, John; Thompson, Alan J; Hunter, Rachael; Pavitt, Sue; Pearson, Owen; Evangelou, Nikos; Sharrack, Basil; Galea, Ian; Chandran, Siddharthan; Ford, Helen; Frost, Chris; Chataway, Jeremy

VERSION 1 - REVIEW

**REVIEWER NAME**	Camara-Lemarroy, Carlos R
**REVIEWER AFFILIATION**	University of Calgary
**REVIEWER CONFLICT OF INTEREST**	None related to this manuscript
**DATE REVIEW RETURNED**	15-May-2024

**GENERAL COMMENTS**	Interesting trial design, addressing a major need in the field- the management of progressive MS.<br><br>It seems data collection will be complete soon (june-july), which is quite close to the guidelines established by BMJ.<br><br>The primary outcome is "confirmed disability progression at 6 monthly intervals, measured as change from EDSS baseline score". But at points in the design, it seems there is an outcome of time to reaching outcome and another of reaching outcome or not (or perhaps the number of outcome events). Clarity would be welcome.<br><br>There is a section of addressing challenges associated to the COVID pandemic, this is a much needed section and is well explained. Was this the main reason for a smaller recruitment?<br><br>A main inclusion criteria is that "progression rather than relapse must be the major cause of increasing disability in the preceding 2

	years". This leaves several gaps. For example, what is the disability in the past 2 years based on? EDSS change? if so, of how much and what components of the EDSS? Is variability in EDSS allowed? (so an increase in year 1 and a decrease in year 2). How is this 2 year history established, by review or by treating neurologist? What is the definition of steady vs non steady progression? These are non trivial questions, as to define a "classic" progressive phenotype.<br><br>Participants are allowed with recent relapses, but 3 months is given as a timeline. What is the rationale of 3 months, if relapses can take up to a year to recover from?<br><br>In the medications listed as exclusion, I suppose monoclonal antibodies include B cell depleters?. In the treatment allocation, it seems participants taking siponimod would be allowed?<br><br>It is mentioned that EDSS can be done remotely by phone. When is this going to happen, how often, and what are the expected challenges of not capturing all aspects of EDSS?<br><br>In regards to the extension, where does the calculation for sample size come from to establish the chance of progression after 36 months of stability? is this not self selecting for a very mild phenotype of SPMS?<br><br>In calculating sample size, again, where does the 40% worsening, 59% worsening etc. come from? what datasets or registries?<br><br>In page 7, it is mentioned that statins affect astroglial and vascular responses, but in what context?<br><br>A section on analysis of active vs non active SPMS, and on-trial management of relapses or new MRI lesions could be informative.

**REVIEWER NAME**	Traboulsee, Anthony
**REVIEWER AFFILIATION**	University of British Columbia Faculty of Medicine
**REVIEWER CONFLICT OF INTEREST**	None
**DATE REVIEW RETURNED**	06-Jun-2024

**GENERAL COMMENTS**	Some hopefully helpful suggestions<br>Introduction: simvastatin is lipophilic and I believe crosses the BBB. Might be worth adding this (with reference) as further rationale for possibility of impact on SPMS as drug should be able to get to astrocytes as putative target.<br><br>Methods: - MRI is mentioned in the appendix 1 (item 4) and should be included in the methods (personal bias). This may end up being an important supportive outcome if there is an impact on brain atrophy (which has its limitations as a biomarker, but worth doing). Likewise details on the biomarker and OCT subsidies (at least briefly if word count allows)<br><br>Methods: It wasn't clear to me if hypercholesterolemia was assessed during screening for the study. Once enrolled, will participants and clinical trial team be blinded from cholesterol testing results due to risk of unblinding and potential bias?

	Methods: perhaps a comment on how EDSS assessment be standardized? Which version is being used? Will there be a two physician approach (blinded EDSS evaluator and treating physician) though I appreciate that might not be practical from a funding perspective.<br><br>Methods: Is data captured electronically, such as Redcap, and is there an audit plan?<br><br>When was the first participant enrolled? this should be stated in my opinion along with the anticipated enrolment completion date (July 2024 I believe)<br><br>The stats are a bit thin in details but adequate. Any there any plans for a futility analysis if there are too many drop outs or not enough progression events?<br><br>might be nice if the protocol was available

VERSION 1 – AUTHOR RESPONSE

Reviewer 1

Reviewer question: Interesting trial design, addressing a major need in the field- the management of progressive MS.

Author response: Thank you; it is encouraging that the work proposed here is considered salient and useful.

Reviewer question: It seems data collection will be complete soon (june-july), which is quite close to the guidelines established by BMJ.

Author response: The trial timelines have been added to the Methods section, as per the Editor's request (discussed earlier in this document). Data lock has yet to occur, which the team understand brings this manuscript within scope for BMJ Open's policies.

Reviewer question: The primary outcome is "confirmed disability progression at 6 monthly intervals, measured as change from EDSS baseline score". But at points in the design, it seems there is an outcome of time to reaching outcome and another of reaching outcome or not (or perhaps the number of outcome events). Clarity would be welcome.

Author response: The analysis will be performed by time to event analysis (survival analysis), which considers whether event has occurred before a given timepoint. We have clarified in the Primary Outcome section that we only consider time to first progression event rather than the number of progression events.

Reviewer question: There is a section of addressing challenges associated to the COVID pandemic, this is a much needed section and is well explained. Was this the main reason for a smaller recruitment?

Author response: Thank you; the team appreciate the feedback that the Covid section was useful in the paper. Covid challenges were the primary reason for the smaller than originally planned recruitment, yes.

Reviewer question: A main inclusion criteria is that "progression rather than relapse must be the major cause of increasing disability in the preceding 2 years". This leaves several gaps. For example, what is the disability in the past 2 years based on? EDSS change? if so, of how much and what components of the EDSS? Is variability in EDSS allowed? (so an increase in year 1 and a decrease in year 2). How is this 2 year history established, by review or by treating neurologist? What is the definition of steady vs non steady progression? These are non trivial questions, as to define a "classic" progressive phenotype.

Author response: As per Table 2, patients must have a confirmed diagnosis of MS that has entered the secondary progressive stage. Steady progression rather than relapse must be the major cause of increasing disability in the preceding 2 years. Progression can be evident from either an increase of at least 1 point if EDSS baseline score <6, or an increase of 0.5 point if baseline EDSS score is ≥6, or clinical documentation of increasing disability. The clinical documentation will be reviewed by a treating neurologist.

Reviewer question: Participants are allowed with recent relapses, but 3 months is given as a timeline. What is the rationale of 3 months, if relapses can take up to a year to recover from?

Author response: The plan proposed for the MS-STAT2 trial is congruent with the phase 2 MS-STAT and the EXPAND trial designs. Subsequent analysis of the MS-STAT trial showed that this was indeed a non-relapsing SPMS population (c.1-2% of patients p.a. had relapse in trial).

Reviewer question: In the medications listed as exclusion, I suppose monoclonal antibodies include B cell depleters?. In the treatment allocation, it seems participants taking siponimod would be allowed?

Author response: Yes, those on B-cell depletion therapy will be excluded (Table 2, Exclusion Criteria, section 12).

Yes, participants could either commence trial on Siponimod or start use mid-trial, as per UK practice. This has been clarified in the Recruitment section.

Reviewer question: It is mentioned that EDSS can be done remotely by phone. When is this going to happen, how often, and what are the expected challenges of not capturing all aspects of EDSS?

Author response: Yes, the use of tele-EDSS has an option throughout the various iterations of the trial protocol, but its use became more salient for in-person visit attendance challenges with the onset of the Covid-19 pandemic. The Trial Schedule has been clarified to note that it may also be used where participant logistics necessitate it (i.e. in cases where the patient would otherwise have not attended clinic with the data point lost). The trial team reviewed the Lechner-Scott et al. (2003) paper on comparing telephone vs in-person EDSS assessment prior to progressing this strategy. The paper also shows that a telephone EDSS is more comparable to in-person assessment for those with EDSS scores ≥4.0 (entry criteria).

Reviewer question: In regards to the extension, where does the calculation for sample size come from to establish the chance of progression after 36 months of stability? is this not self selecting for a very mild phenotype of SPMS?

Author response: We thank the reviewer for noting this important point. However, no further calculation was done in support of this as it was assumed the progression rate would be the same as during the first 36 months.

Reviewer question: In calculating sample size, again, where does the 40% worsening, 59% worsening etc. come from? what datasets or registries?

Author response: It assumes that a progression rate in the placebo group of 8% per six months in those that have not yet experienced progression. These data are based on a review of previous phase 3 trials in SPMS (Ontaneda et al. 2015), as well as two papers reporting out Interferon beta in SPMS (Anderson et al. 2004; Panitch et al. 2004) and one paper evaluating cannabinoid use in SPMS (Ball et al. 2015). These references and the progression rate have been added to the Sample Size section.

Reviewer question: In page 7, it is mentioned that statins affect astroglial and vascular responses, but in what context?

Author response: We refer the reviewers to the most recent review (2020) by Bagheri et al., but in essence the review demonstrated that a growing body of animal and cellular evidence has indicated that statins have the potential to attenuate the proinflammatory mediators and subsequent neurodegenerative disorders by controlling the microglial activation and consequent reduction in neuroinflammatory mediators.

Reviewer question: A section on analysis of active vs non active SPMS, and on-trial management of relapses or new MRI lesions could be informative.

Author response: As this is a protocol paper, we are not able to add this into the project as it is not included in the current approved protocol. However, we note that there is a separately funded sub-study on MRI outcomes in this population, including analysis of new and enlarging MRI lesions. In the results papers we will explore these issues.

Reviewer 2

Reviewer question: Introduction: simvastatin is lipophilic and I believe crosses the BBB. Might be worth adding this (with reference) as further rationale for possibility of impact on SPMS as drug should be able to get to astrocytes as putative target.

Author response: We thank the reviewer for this useful comment. An existing citation (Bagheri et al., 2020) contains a reference to Wood et al. Statins and Neuroprotection (2010), which speaks in more detail to this point.

Reviewer question: Methods: MRI is mentioned in the appendix 1 (item 4) and should be included in the methods (personal bias). This may end up being an important supportive outcome if there is an impact on brain atrophy (which has its limitations as a biomarker, but worth doing). Likewise details on the biomarker and OCT subsidies (at least briefly if word count allows)

Author response: Yes, additional substudies investigating MRI, biomarker and OCT outcomes will be carried out at the lead site only (University College London Hospitals Trust) for MS-STAT2 participants. As they will be reported separately to the main trial results, details for them were not initially provided in this manuscript. A supporting note has now been added around this to the Trial Schedule.

Reviewer question: Methods: It wasn't clear to me if hypercholesterolemia was assessed during screening for the study. Once enrolled, will participants and clinical trial team be blinded from cholesterol testing results due to risk of unblinding and potential bias?

Author response: The protocol mandates that a lipid profile is completed at baseline. This has been clarified in the Recruitment section. Once enrolled, no further trial-specific assessments of lipids are requested to avoid unblinding participants.

Reviewer question: Methods: perhaps a comment on how EDSS assessment be standardized? Which version is being used? Will there be a two physician approach (blinded EDSS evaluator and treating physician) though I appreciate that might not be practical from a funding perspective.

Author response: Yes, EDSS assessment will be performed by a blinded assessor at each site, and work to the current version of the Neurostatus scoring guidance (v04/10.2). This has been clarified in the Primary Outcome section.

Reviewer question: Methods: Is data captured electronically, such as Redcap, and is there an audit plan?

Author response: Data are initially recorded on paper CRFs, with site data entry to an Elsevier MACRO v4.0 database. This has been clarified in the Data Management section. Yes, there is a site activity and data quality monitoring (audit) plan, which is updated at least annually and reviewed by our Clinical Trials Unit's Quality group – further detail on this process has been added to the Audit section.

Reviewer question: When was the first participant enrolled? this should be stated in my opinion along with the anticipated enrolment completion date (July 2024 I believe)

Author response: The first participant was randomised into the trial in May 2018. The trial timelines have now been added to the Methods section, as per the Editor's request (discussed earlier in this document).

Reviewer question: The stats are a bit thin in details but adequate. Any there any plans for a futility analysis if there are too many drop outs or not enough progression events?

Author response: There are several interim analyses for safety and efficacy in the protocol which were not initially included due to word limit. Additionally, the IDMC would consider stopping the trial for futility on the basis of recruitment figures. These have now been added into a new sub-section called Interim Analyses within the Analyses section.

Reviewer question: might be nice if the protocol was available

Author response: We agree with the reviewer and anticipate making the protocol available as an attachment to the primary results paper.
